# Defining the expression hierarchy of latent T-cell epitopes in Epstein-Barr virus infection with TCR-like antibodies

**DOI:** 10.1038/srep03232

**Published:** 2013-11-18

**Authors:** Adrian Chong Nyi Sim, Chien Tei Too, Min Zin Oo, Junyun Lai, Michelle Yating Eio, Zhenying Song, Nalini Srinivasan, Diane Ai Lin Tan, Shyue Wei Pang, Shu Uin Gan, Kok Onn Lee, Thomas Kwok Seng Loh, Jianzhu Chen, Soh Ha Chan, Paul Anthony MacAry

**Affiliations:** 1 Immunology Program, Department of Microbiology, Yong Loo Lin School of Medicine, National University of Singapore, Singapore 117456, Singapore; 2 NUS Graduate School of Integrative Sciences and Engineering (NGS), National University of Singapore, Singapore 117456, Singapore; 3 Infectious Disease Interdisciplinary Research Group, Singapore-MIT Alliance for research and technology (SMART), National University of Singapore, Singapore 138602, Singapore; 4 WHO Immunology and Training Research Centre, National University of Singapore, Singapore 117597, Singapore; 5 Department of Surgery, Yong Loo Lin School of Medicine, National University of Singapore, Singapore 119228, Singapore; 6 Defence Medical and Environment Research Institute, DSO National Laboratories, Singapore 118230, Singapore; 7 Department of Otolaryngology (ENT), National University Hospital, Singapore 119228, Singapore; 8 Department of Medicine, National University Hospital, Singapore 119228, Singapore; 9 These authors contributed equally to this work.

## Abstract

Epstein-Barr virus (EBV) is a gamma herpesvirus that causes a life-long latent infection in human hosts. The latent gene products LMP1, LMP2A and EBNA1 are expressed by EBV-associated tumors and peptide epitopes derived from these can be targeted by CD8 Cytotoxic T-Lymphocyte (CTL) lines. Whilst CTL-based methodologies can be utilized to infer the presence of specific latent epitopes, they do not allow a *direct* visualization or quantitation of these epitopes. Here, we describe the characterization of three TCR-like monoclonal antibodies (mAbs) targeting the latent epitopes LMP1_125–133_, LMP2A_426–434_ or EBNA1_562–570_ in association with HLA-A0201. These are employed to map the expression hierarchy of endogenously generated EBV epitopes. The dominance of EBNA1_562–570_ in association with HLA-A0201 was consistently observed in cell lines and EBV-associated tumor biopsies. These data highlight the discordance between MHC-epitope density and frequencies of associated CTL with implications for cell-based immunotherapies and/or vaccines for EBV-associated disease.

EBV is a persistent herpesvirus acquired as a predominantly asymptomatic infection during childhood in most human communities^1^. The virus can infect cells of both lymphoid and epithelial origin and its latent infection phase is associated with malignancies that arise from these cell types, including Non-Hodgkin's lymphoma, Hodgkin's Lymphoma^2^ and undifferentiated nasopharyngeal carcinoma (NPC)^3^. EBV latent gene products found in tumors include Epstein-Barr Virus Nuclear Antigen 1 (EBNA1) and/or Latent Membrane Protein 2A (LMP2A) and/or Latent Membrane Protein 1 (LMP1) depending upon the latency program employed by the virus^1^,^2^. Despite the subdominant frequencies of CTLs specific for epitopes derived from these latent gene products (0.05%–1%), they are implicated in the control of EBV infection *in vivo*^1^. The presence of these gene products in the majority of EBV-associated tumors suggests that an analysis of their associated CTL epitopes is essential for the design of immuno-targeting approaches including adoptive T-cell therapy.

The expression of EBV-latent epitopes in infected cells has been inferred *indirectly* from studies employing CTLs, HLA tetramer analysis, and targeted lysis by T-cell lines^1^. Notably however, T-cell functionality is pivotal for these analyses, and antigen specific CTLs can be rendered dysfunctional by viral immune evasion mechanisms^4^. A *direct* measure of HLA-peptide epitopes would circumvent this problem.

The direct analysis of surface EBV latency epitopes presented on MHC class I can be determined using mass spectrometry but this is highly dependent on their hydrophobicity and ionization potential^5^.

An optimal approach is to develop antibodies that recognize viral epitopes in association with MHC^6^. Termed TCR-like mAbs, these reagents exhibit high affinities and enable direct visualization and quantification of the specific epitope presented^7^. In this study, antibodies targeting epitopes of EBV latent gene products (LMP1_125–133_, LMP2A_426–434_ and EBNA1_562–570_) were generated and characterized. This allowed an analysis of viral epitope expression using a combination of immunological and biochemical methods including flow cytometry, immunohistochemical staining, and confocal microscopy. We next established the epitope expression hierarchy amongst the three latent epitopes in cell lines and clinically relevant EBV-associated tumor biopsies. Our observations of this hierarchy and its differential binding on strain-associated epitope variants have important implications for diagnosis, immuno-targeting and vaccine development.

## Results

### Generation of high affinity TCR-like mAbs with exquisite specificity

**Figure 1:**
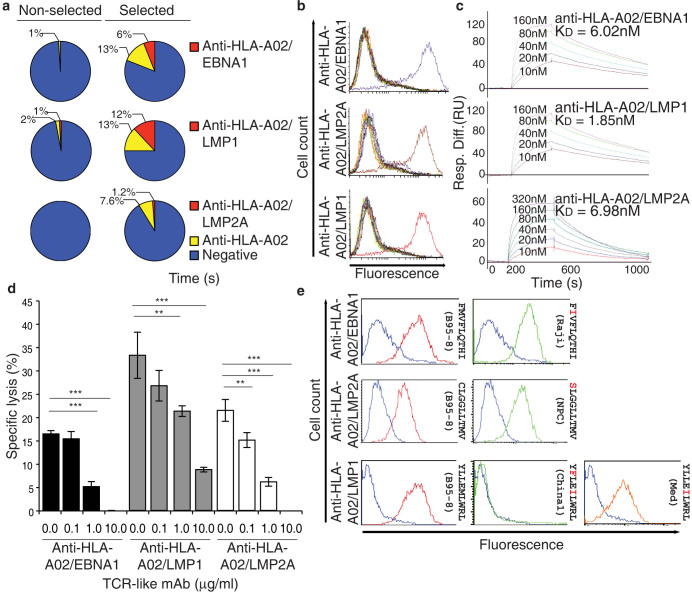
Summary of TCR-like mAbs screened and characterization of TCR-like mAbs. (a) Summary of hybridomas isolated for each of the TCR-like mAbs with and without prior enrichment for specific splenocytes prior to fusion. (b) Fine binding specificity of the TCR-like mAbs to T2 cells pulsed with the EBNA1_562–570_ (purple histogram), LMP1_125–133_ (red), LMP2A_426–434_ (brown), Influenza A M1_58–66_ (dark green), *Mycobacterium tuberculosis* Ag85B_143–152_ (pink), HBV sAg_183–191_ (light blue), HIV Pol_476–484_ (blue), gp120_120–128_ (yellow), Gag_77–85_ (grey), CMV IE1_316–324_ (orange), IE1_81–89_(black), pp65_495–503_ (light brown) or unpulsed cells (light green).(c) Affinity determination of the TCR-like mAbs using surface plasmon resonance by flowing various concentrations of respective HLA-A0201/peptide complexes over CM5 chip-bound TCR-like mAbs. (d) Inhibition of specific lysis by specific CTL using respective TCR-like mAbs at concentrations of 0.1 μg/ml, 1 μg/ml and 10 μg/ml. Error bars are standard deviation from average of three independent experiments: **p < 0.01,***p < 0.005.(e) Differential recognition of the TCR-like mAbs against respective variants of epitopes pulsed on HLA-A0201 positive BLCLs. Blue histogram denotes staining with murine isotype antibody MOPC1.

In this report, we highlight an adaptation of conventional hybridoma technology that enabled the production of high-affinity TCR-like mAbs targeting three EBV latent epitopes displayed on HLA-A0201. The methodology is illustrated in Supplementary Information (Supplementary Fig. S1). Briefly, membrane-free HLA-A0201 associated with EBV latent peptides (EBNA1_562–570_: FMVFLQTHI; LMP1_125–133_: YLLEMLWRL; LMP2A_426–434_: CLGGLLTMV) were generated to immunize mice using an established protocol^8^,^9^,^10^,^11^. The splenocytes of immunized mice were immuno-magnetically selected prior to fusion. It is only with this enrichment that hybridomas producing TCR-like mAbs targeting EBNA1_562–570_ and LMP2A_426–434_ in association with HLA-A0201 can be generated (Fig. 1a). For hybridomas producing antibodies targeting LMP1_125–133_ in association with HLA-A0201, there is an increase in the percentage of such hybridomas isolated following splenocytes enrichment. The optimal representative monoclonal hybridoma for each target was selected for subsequent analyses.

The binding specificities of these antibodies were examined using flow cytometric analysis of T2 cells pulsed with 12 different HLA-A0201 restricted peptides. The mAbs exhibited exquisite specificity for their respective target peptide and not other HLA-A0201 restricted epitopes from a variety of human pathogens (Fig. 1b).

To determine the binding affinities of the three antibodies, surface plasmon resonance (SPR) was employed. All three mAbs exhibited strong binding affinities for their respective ligands (anti-HLA-A02/EBNA1_562–570_ K_D_ = 6.02 nM; anti-HLA-A02/LMP1_125–133_ K_D_ = 1.85 nM; anti-HLA-A02/LMP2A_426–434_ K_D_ = 6.98 nM) (Fig. 1c).

To further examine the specificity of each TCR-like mAb in comparison to specific CTL, we determined their ability to inhibit CTL lysis. The three mAbs inhibited the activity of their respective CTLs in a specific dose-dependent manner, as shown by the inhibition of CTL-inflected ^51^Cr release from target cells (Fig. 1d). Thus, we can infer a degree of overlap in the targeting of TCRs and TCR-like mAbs for the same viral epitopes. With these mAbs endowed with TCR specificity, we can visualize and quantitate the expression profile of latent EBV epitopes in infected cells.

### Epitope variants are differentially recognized by respective TCR-like mAbs

A factor that impacts upon epitope presentation is strain differences in the encoding sequence of the latent antigens that translates to CTL epitope variants. The classical methodology of EBV typing does not adequately distinguish the pathogenic/tumorigenic nature of various virus strains^12^. This typing does not give due consideration to the clinical diversity of EBNA1, LMP1, and LMP2A, which are the only three latent genes observed in associated malignancies^1^. Using alternative classifications that were adopted for each of these genes, we investigated the presentation of clinical epitope variants^9^,^10^,^11^. Each of these epitope variants was pulsed onto HLA-A0201 homozygous B cell lymphoblastoid cell lines for flow cytometric analysis using the three TCR-like mAbs (Fig. 1e). Anti-HLA-A02/EBNA1_562–570_ antibody can bind the B95-8 and Raji variants. Anti-HLA-A02/LMP1_125–133_ antibody exhibited good binding activity for B95-8 and Mediterranean (Med) variants but none was observed for cells pulsed with the China1 variant-the form of EBV that is linked to NPC^10^. Strong binding was observed for anti-HLA-A02/LMP2A_426–434_ antibody for both the B95-8 and the NPC variants. With the exception of the LMP1 (China1) variant, our TCR-like mAbs can recognize the clinical variants of our epitopes of interest (Fig. 1e). Together, these findings indicate that epitopes associated with HLA-A0201 on infected cells are differentially presented and this can be influenced by mutations associated with strain variation.

### Mapping the expression hierarchy of three latent epitopes on EBV infected cells

**Figure 2:**
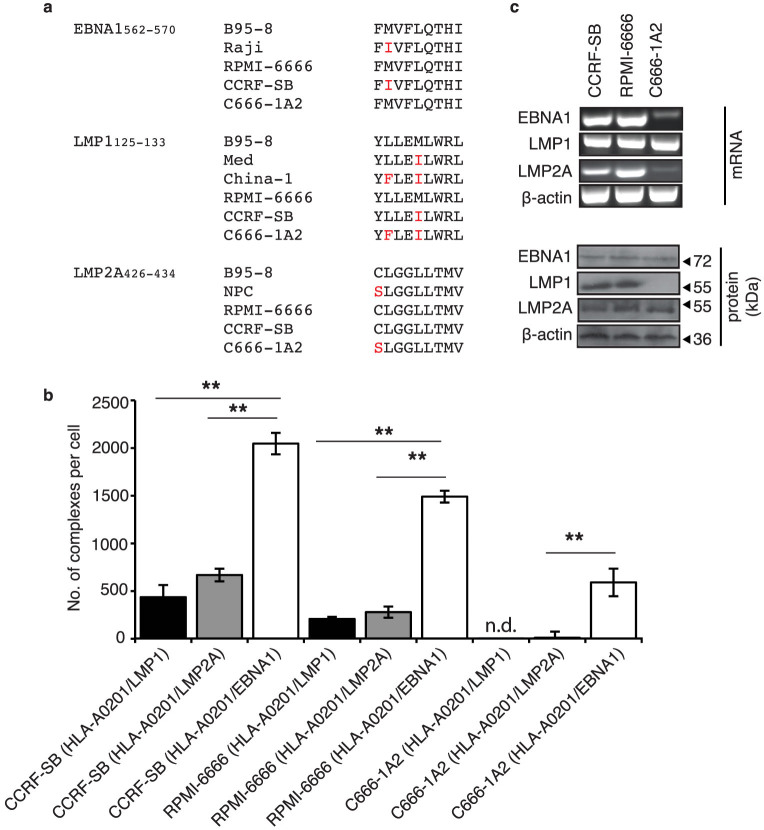
Expression hierarchy of latent EBV epitopes in infected HLA-A0201 cell lines. (a) Epitope comparison amongst the variants and those observed in the 3 cell lines studied. Red letters denote amino acid mutation. (b) Quantitation of HLA-A0201/LMP1,/LMP2A and/EBNA1 on CCRF-SB, RPMI-6666 and C666-1A2 using TCR-like mAbs based on flow cytometry. Error bars are standard deviation from an average of three independent experiments. **p < 0.01. n.d. denotes not detectable. (c) LMP1, LMP2A and EBNA1 in each of the cell lines was determined using RT-PCR and Western blot. β-actin was used as control. The blots and gels were cropped from full-length blots/gels presented in Supplementary Figure S3.

We next analyzed the endogenous expression of EBNA1_562–570_, LMP1_125–133_ and LMP2A_426–434_ epitopes on three HLA-A0201 EBV-infected tumor cell lines of lymphoid and epithelial origins: CCRF-SB, RPMI-6666 and C666-1A2^13^. The epitope variant of each cell line was determined by sequencing their associated EBV virus (Fig. 2a). A flow cytometry based bead-calibration kit, QIFIKIT® (Dako), allowed the quantitation of the three EBV latent peptides presented by HLA-A0201 (Supplementary Fig. S2). This enabled us to map the ligand density and expression hierarchy amongst the three latent EBV epitopes in the cell lines (Fig. 2b). HLA-A0201/EBNA1_562–570_ complexes were strongly expressed by the RPMI-6666 and CCRF-SB cell lines (1500 ± 61 and 2050 ± 112 complexes respectively). An intermediate level of expression was observed on the C666-1A2 cell line (590 ± 144.3 complexes). In contrast, HLA-A0201/LMP1_125–133_ and/LMP2A_426–434_ complexes exhibited relatively intermediate or weak expression on CCRF-SB (437 ± 127 and 668 ± 66 complexes respectively) and RPMI-6666 (208 ± 22 and 280 ± 59 complexes respectively) and negligible complexes on C666-1A2. This represents the first analysis of an expression hierarchy of HLA-A0201/peptide complexes of latent EBV antigens. Surprisingly, the high expression and dominance of HLA-A0201/EBNA1_562–570_ complexes was consistent for all cell lines tested. With the exception for the LMP1 (China1) variant of C666-1A2, our mAbs could bind the variants of each epitope. This was consistent with our previous data on binding of epitope variants (Fig. 1e).

The mAbs were also utilized to determine the cellular localization and relative expression of the 3 latent EBV HLA-A0201/peptide complexes by confocal microscopy. A high degree of surface localization of the HLA-A0201/EBNA1_562–570_ complexes was observed in all three cell lines (Supplementary Fig. S4) and the staining intensities of the three latent antigens correlated with the quantitation data shown in Figure 2b. In all cases, expression of HLA-A0201/EBNA-1_562–570_ complexes on the surface of the tumor cells was stronger than/LMP-1_125–133_ or/LMP-2A_426–434_. Our observations cannot be ascribed to the presence or absence of the latent proteins from which the epitopes were derived, as indicated by RT-PCR and immunoblot (Figure 2c). The absence of an LMP1 immunoblot for C666-1A2 was due to the mutations in the LMP1 (China1) variant that altered the binding site for the anti-LMP1 antibody used^13^.

### Detection of latent viral epitope expression in a humanized EBV mouse model

To analyze the expression of the three antigens in the context of a natural EBV infection, we employed an EBV-infected humanized NOD/SCID/IL2rγ^null^ (Hu-NSG) mouse model based on reported protocols^14^. The latency program exhibited by these mice was type III and thus the same as that observed in lymphoproliferative disorders in immuno-compromised patients^1^.

**Figure 3:**
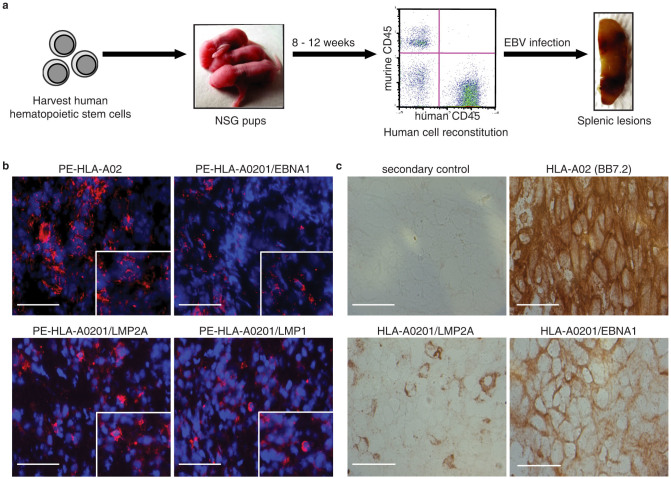
Generation of Hu-NSG mice EBV model and staining of infected mouse spleen and clinical NPC biopsies with TCR-like mAbs. (a) Experimental workflow of the Hu-NSG mice EBV model -isolation of HSC; injection into NSG mice; flow cytometric analysis of reconstitution; EBV infection of Hu-NSG mice; and development of splenic lesions. The reconsitution of the Hu-NSG mice with human immune cells was approximately 65% ± 3.9%. (b) Spleens of EBV infected HLA-A0201 Hu-NSG mice showed positive red staining for TCR-like mAbs specific to LMP1, LMP2A and EBNA1 binding. Scale bars, 50 μm. (c) Immunological staining of NPC biopsy. Control panel was stained with secondary antibody. BB7.2 antibody stained for HLA-A02. The other two panels were stained with anti-HLA-A0201/LMP2A and anti-HLA-A0201/EBNA1 mAb. Positive staining was observed to be reddish brown. Scale bars, 25 μm.

A schematic of our experimental approach is provided (Fig. 3a). Following hematopoietic stem cells (HSC) reconstitution in NSG mice and infection with EBV, the spleens were harvested for staining. Immunofluorescence staining was carried out using R-Phycoerythrin (PE) conjugated TCR-like mAbs (Fig. 3b). Due to the haplotype specific nature of our mAbs, only mice engrafted with HLA-A0201 HSC exhibited staining for all three complexes (Fig. 3b). Thus, these staining validated the employment of our TCR-like mAbs for epitope presentation studies in EBV-infected Hu-NSG mouse model.

### Dominance of EBNA1_562–570_ epitope expression in EBV-tumor biopsies

To determine the clinical relevance of our observations, cryo-sections of HLA-A0201 EBV-positive NPC biopsies were stained with the respective TCR-like mAbs. EBV infected cells in these biopsies stained strongly for HLA-A0201/EBNA1_562–570_ complexes and weakly for HLA-A0201/LMP2A_426–434_ complexes. Sections were also stained with BB7.2 to determine their expression of HLA-A02 molecules. A representative data set (from 5 patients) is shown (Fig. 3c). The staining intensity correlated with that observed in the NPC cell line C666-1A2 (Supplementary Fig. S3). The LMP1_125–133_ complex staining was not observed due to epitope mutation in the EBV NPC-linked China1 strain, correlating with the data in Figure 1e.

## Discussion

TCR-like mAbs are valuable reagents for direct epitope visualization and detection. Several studies had illustrated its usefulness in epitope discovery^15^, epitope processing^16^, and immuno-targeting^17^. However, the numbers of documented antibodies are limited.

Previous reports documenting the generation of these mAbs used either classical hybridoma fusion following murine immunization or phage display methodologies. These resulted in antibodies that are relatively low in affinity^18^ and stain poorly for endogenously generated epitopes^19^.

By enriching for immunized splenocytes prior to fusion, we increased the number of hybridomas producing antibodies targeting LMP1 compared to non-enrichment. Moreover, it is only with this enrichment approach that hybridomas producing two of our TCR-like mAbs targeting EBNA1 and LMP2A could be isolated. Futhermore, our antibodies exhibit high specificity for their respective epitopes and binding affinity in the nanomolar range. Our data validates this approach for isolating such rare hybridomas^5^ that are technically challenging to generate^20^,^21^.

The high affinity and exquisite specificity of these reagents enabled us to examine epitope variants presented on HLA-A0201 BLCLs. These epitope mutations are due to viral sequence variation observed in each of the latent EBV genes (LMP1, LMP2A and EBNA1). Our antibodies bind to the epitope variants with differential activity suggesting that the associated amino acid substitutions impact upon presentation. Given that B95-8 is the only GMP-grade EBV strain employed for the expansion of specific CTL for adoptive immunotherapy, our data indicate that more attention must be paid to the EBV strain infecting the patient to ensure *in vivo* efficacy of the adoptively transferred T- cells.

Staining for *endogenously* produced epitopes/peptides is a major challenge for TCR-like mAbs^19^. Therefore, it was necessary to investigate the binding ability of our mAbs for its respective endogenously generated cognate peptide/MHC. As observed, our reagents enabled the visualization of intracellular and extracellular localization of endogenously generated epitopes. These reagents were also used to visualize latent EBV epitope expressing cells in an EBV-infected humanized mouse model. Coupled with QIFIKIT®, we also quantitated the density of endogenously generated epitopes in these three EBV infected cell lines. A distinct epitope expression hierarchy was consistently observed for all three cell lines with the dominance of EBNA1_562–570_ over the other two epitopes analyzed. This was similarly observed in clinically relevant EBV-associated NPC biopsies. The dominance of EBNA1_562–570_ epitope did not correlate with the reported frequency of the respective specific CTLs^22^ where CTLs targeting LMP2A_426–434_ are dominant over the other two epitopes (EBNA1_562–570_ and LMP1_125–133_).

Previous reports have intimated that EBNA1 inhibits its own processing and presentation^23^. However, epitope expression can be inferred from the presence of EBNA1 specific CTLs-this has been proposed as evidence for dendritic cells cross-presentation of EBNA1 peptides^24^. Despite such indications, we are the first to directly visualize and detect HLA-A0201/EBNA1_562–570_ complex expression in infected cells of both hematopoietic and non-hematopoietic origin. This suggests that EBNA1 can be processed and presented through the classical MHC class I pathway. Although the presentation of HLA-A0201/EBNA-1_562–570_ was reported to be low or non-existent in previous reports employing CTL lines^21^,^25^,^26^, we were able to *directly* detect this “rare” epitope in relatively high numbers. This discordance could be due to dysfunctional CTLs^4^ or suggest that the density of peptide-MHC complexes is not necessarily the key factor in CTL selection and/or dominance. The immunodominance of CTLs can be affected by an interplay of cellular and molecular factors such as CTL precursor frequencies^27^, quality of TCR-peptide-MHC signaling potency^28^ and the relative binding affinity of the peptide epitope for its associated MHC molecule^29^. These components and *in vivo* tumor microenvironment^4^ can influence both the scale and form of the CTL response but were not addressed as part of this study.

In summary, by employing TCR-like mAbs, we have determined the expression hierarchy of EBV latent antigens. Our data indicates that EBNA1_562–570_ and LMP2A_426–434_ are possible biomarkers of EBV-associated tumors in HLA-A0201 patients. The dominance of EBNA1_562–570_ amongst the three epitopes was validated in cell lines and *ex vivo* staining of clinical NPC biopsies. This observation has important implications for both immunotherapy and EBV vaccine development.

## Methods

### Cell lines

The cell lines RPMI-6666 (CCL-113, Hodgkin's lymphoma), CCRF-SB (CCL-120, acute lymphoblastic leukemia) and T2 (CRL-1992, TAP negative lymphoblast) were obtained from the American Tissue Culture Collection. C666-1A2 is a NPC cell line^13^ transduced with HLA-A0201 haplotype. The B cell lymphoblastoid cell lines were generated from donors.

### Antibody and peptide

The HLA-A02-restricted peptides Epstein-Barr virus EBNA1 (B95-8)_562–570_ (FMVFLQTHI), EBNA1 Raji variant (FIVFLQTHI), LMP1 (B95-8)_125–133_ (YLLEMLWRL), LMP1 China1 variant (YFLEILWRL), Med variant (YLLEILWRL), LMP2A(B95-8)_426–434_ (CLGGLLTMV), LMP2A NPC variant (SLGGLLTMV), *Mycobacterium tuberculosis* Ag85B_143–152_ (FIYAGSLSAL), Hepatitis B virus sAg_183–191_ (FLLTRILTI), HIV Pol_476–484_ (ILKEPVHGV), gp120_120–128_ (KLTPLCVTL), Gag_77–85_ (SLYNTIAVL), CMV IE1_316–324_ (VLEETSVML), IE1_81–89_ (VLAELVKQI), pp65_495–503_ (NLPVMVATV) and Influenza A virus M1_58–66_ (GILGFVFTL) were synthesized by Mimotopes to 95% purity and was analyzed by electrospray mass spectrometry. The murine IgG1 isotype control antibody MOPC 21 was purchased from Sigma-Aldrich. The rabbit anti-beta-2 microglobulin polyclonal antibody and horseradish-peroxidase-conjugated goat anti-mouse IgG were purchased from Thermo Fisher Scientific. CS1-4 antibody cocktail (anti-EBV LMP1 antibody) and PE-conjugated goat anti-mouse IgG (H + L) were purchased from Dako. Alexa Fluor® 488-conjugated goat anti-mouse IgG (H + L) antibodies were purchased from Invitrogen. 14B7 (anti-EBV LMP2 antibody) and E1-2.5 (anti-EBV EBNA1 antibody) were purchased from Abd Serotec. Anti-human CD20 antibody was purchased from Abcam.

### Surface plasmon resonance (SPR)

Kinetic studies were evaluated by surface plasmon resonance using a BIAcore 3000™ (GE Healthcare). The monoclonal antibodies were immobilized covalently onto the surface of sensor chip CM5 by amine coupling. To determine the dissociation constant (K_D_) of the antibodies, the respective MHC class I/peptide were flowed at different concentrations ranging from 320 nM to 10 nM over the relevant wells at a rate of 30 μL min^−1^ at 25°C. Responses were recorded and analyzed using BIAevaluation software 3.2.

### Western blot

CCRF-SB, RPMI666, and C666-1A2 cells were cultured in 100 mm dishes and lysed with 1 × RIPA buffer (Thermo Fisher Scientific) supplemented with protease inhibitors (Roche Applied Science). The lysates were boiled for 15 min and separated by 10% SDS-polyacrylamide gel electrophoresis, and transferred onto polyvinylidene difluoride membrane (GE Healthcare) overnight. The membranes were probed with CS1-4 antibody cocktail (anti-EBV LMP1 antibody) (1:2000 dilution) (Dako), 14B7 (anti-EBV LMP2A antibody) (1:5000 dilution) (AbD Serotec) or E1-2.5 (anti-EBV EBNA1 antibody) (1:5000 dilution) (AbD Serotec). The membranes were subsequently incubated with horseradish-peroxidase-conjugated goat anti-mouse IgG or goat anti-rat IgG (Thermo Fisher Scientific). The proteins were detected with enhanced chemiluminescence reagent (Western Lightning ECL kit, Perkin Elmer).

### Detection of LMP1, LMP2A and EBNA1 transcripts by RT-PCR

RNA from 1 × 10^6^ cells was extracted using High Pure RNA Isolation kit (Roche Diagnostics) and 1 μg of total RNA was amplified by RT-PCR with OneStep RT-PCR kit (Qiagen) using the appropriate primers as previously reported^30^. Beta actin was used as an internal control.

### Quantitation assays

Epstein-Barr virus infected HLA-A02 cells were stained with the various TCR-like mAb followed by PE-conjugated goat-anti-mouse IgG (Dako). The level of fluorescence intensity was compared with that of calibrated beads containing pre-determined quantity of mouse monoclonal antibody per bead (QIFIKIT® calibration beads; Dako). By means of a standard curve based upon the calibration beads, the number of complexes on the cell surface was determined from the fluorescence intensity of each TCR-like monoclonal antibody.

### Raising of specific CTL lines

The HLA-A0201 restricted CTL specific for peptides of Epstein-Barr associated proteins – LMP1_125–133_ (YLLEMLWRL), LMP2A_426–434_ (CLGGLLTMV) or EBNA1_562–570_ (FMVFLQTHI) was prepared. CD8+ T cells were isolated from a healthy HLA-A0201 positive donor. Human peripheral blood mononuclear cells (PBMC) were isolated from fresh heparinized blood from healthy donor by Ficoll-Paque density gradient centrifugation and CD8+ T cells were subsequently isolated using MACs isolation of CD8+ positive T cells (Miltenyi Biotec) in AIM ® V media supplemented with 2% human AB serum. These isolated CD8 T cells were specifically expanded by adding it into wells seeded with 1 μM of LMP1_125–133_ (YLLEMLWRL), LMP2A_426–434_ (CLGGLLTMV) or EBNA1_562–570_ (FMVFLQTHI) peptides pulsed irradiated PBMC feeder cells and incubated at 37°C in a CO_2_ incubator. On day 2 after isolation, the media of the wells were supplemented with 20 U/ml IL2 to expand specific peptide stimulated CD8+ T cells. On day 10, CTLs were cloned by limiting dilution assay and expanded in AIM ® V media supplemented with 2% AB serum, 20 U/ml IL2, 5 ng/ml IL7 and 100 pg/ml IL12 (R&D Systems) at a density of 500,000 cells/well in 96-well plates seeded with irradiated PBMC feeder cells pulsed with 1 μM of respective specific peptides.

### Confocal microscopy

RPMI-6666 and CCRF-SB were seeded onto poly-L-lysine coated glass coverslips while C666-1A2 was seeded overnight on glass coverslips before fixing with methanol-acetone solution. The fixative was quenched with PBS-10 mM glycine before staining was carried out using the respective TCR-like mAbs or rabbit anti-beta-2-microglobulin polyclonal antibody (Thermo Fisher Scientific). Staining was performed in a humidity chamber for 1 h in permeabilization buffer with the concentrations of antibody at 1:1000 dilutions. The coverslips were subsequently stained with either PE-conjugated goat anti-mouse IgG (H + L) (Dako) or Alexa Fluor® 647-conjugated goat anti-rabbit IgG (H + L) (Invitrogen) with DAPI. ProLong Gold Antifade reagent (Invitrogen) was used to mount the slides prior to analysis with a Leica TCS SP5 confocal microsystems (Leica Microsystems) using the HCX PLAPO 63 × objective (numerical aperture: 1.4) The images were acquired at a resolution of 1024 × 1024 pixels for each set.

### Flow cytometry

Approximately 5 × 10^5^ cells were incubated with mAbs for 1 h at 4°C. After washing with 1 × PBS, the cells were incubated with Alexa Fluor® 488-conjugated goat anti-mouse IgG (H + L) (Invitrogen) for 30 min at 4°C. The samples were assessed with FACSCalibur and analyzed using Cell Quest software (BD Biosciences).

### Chromium-release cytotoxic assay

3 × 10^5^ C1R-A2 cells were incubated with 50 μCi ^51^Cr (Perkin Elmer) in 100 μl culture medium, with or without the relevant peptide (5 μM) for 1 h at 37°C, and plated in 96-well culture plates (3,000 cells per well). HLA-A02 restricted epitope specific CTL lines were then incubated for 3.5 h in 200 μl culture medium at an E/T ratio of 10:1. Supernatant was harvested from each well and transferred onto a lumaplate (Perkin Elmer) for analysis with a TopCount NXT (Perkin Elmer). The percentage of release was calculated using the formula: 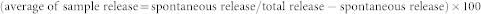


### TCR-like mAb staining of Hu-NSG mouse tissues

Spleens were harvested from EBV-infected Hu-NSG mice and frozen in OCT medium. Tissue cryosections were subsequently prepared on poly-L-lysine coated slides. The sections were fixed and incubated with anti-human and anti-mouse FcR blocking agents (Miltenyi Biotec) to prevent non-specific binding of the antibodies. The tissues were stained with PE-conjugated TCR-like mAbs for 2 h. After washing, each slide was coated with DAPI containing SlowFade Gold solution (Invitrogen). The slides were scanned and viewed with MiraxViewer 1.12 software (Carl Zeiss).

### Immunohistochemistry

Frozen NPC biopsies were sectioned (4 μm) and embedded onto slides. Slides were fixed and subsequently stained with TCR-like mAbs or BB7.2^31^. Peroxidase block (Dako) was used prior to treatment with Dako labeled polymer. After further washing, immunoperoxidase staining was developed with DAB (Dako).

### Animal experiments

BALB/c and NOD/SCID/IL2rγ^null^ (NSG) mice were housed in appropriate facilities. All experiments were approved by the IACUC of the National University of Singapore and conducted in accordance with the Guide for the Care and Use of Laboratory Animals (IACUC no: 027/07 and 106/10).

### Biopsy and blood collection

Informed consent from healthy volunteers and patients were taken before biopsy or blood extraction. NPC patients at the National University Hospital were enrolled with informed consent. The Institutional Review Board of the hospital approved this study (IRB no: 07-043E).

### Statistical analysis

Empirical data were analyzed using GraphPad Prism, version 5.0. 1-way ANOVA with Bonferroni post-test.

## Author Contributions

 A.C.N.S. performed most of the experiments. C.T.T., M.Y.E., and N.S. generated the hybridomas. D.A.L.T. and S.U.G. generated the C666-1A2 cell line. S.W.P. sequenced and analyzed the epitopes. Z.S. stained the biopsies. M.Z.O. assisted with the mouse experiments. J.L. performed the *in vitro* experiments. K.O.L., T.K.S.L., J.C. and S.H.C. shared expertise and materials. A.C.N.S. and P.A.M. designed all experiments, analyzed the results, and wrote the manuscript.

## Supplementary Material

Supplementary Information Supplementary material and supplementary figures
