# Supplementary material for: Defining the expression hierarchy of latent T-cell epitopes in Epstein-Barr virus infection with TCR-like antibodies
Source: Sci Rep. 2013 Nov 18;3:3232. doi: 10.1038/srep03232 (PMC3831236; doi:10.1038/srep03232)
Supplement: Supplementary Information — Supplementary material and supplementary figures [file srep03232-s1.pdf]

Defining the expression hierarchy of latent T-cell epitopes in Epstein-Barr virus infection with TCR-like antibodies.

Adrian Chong Nyi Sim[1,2,*], Chien Tei Too[1,*], Min Zin Oo[3], Junyun Lai[1], Michelle Yating Eio[1], Zhenying Song[1], Nalini Srinivasan[4], Diane Ai Lin Tan[5], Shyue Wei Pang[6], Shu Uin Gan[5], Kok Onn Lee[8], Thomas Kwok Seng Loh[7], Jianzhu Chen[3], Soh Ha Chan[4] and Paul Anthony MacAry[1,2]

Supplementary

Methods

Refolding HLA Class I Molecules *in vitro*

HLA-A0201-beta-2 microglobulin complexes were refolded with the peptide epitopes from EBNA1, LMP1 and LMP2A as follows: 8 mg of beta-2 microglobulin were mixed with 12 mg of the purified HLA-A0201 heavy chain and 6 mg of peptide with 1 ml of guanidine/acetate solution (3 M guanidine-hydrochloride, 10 mM sodium acetate, 10 mM Na-EDTA, 0.5 M EDTA). The mixture is then pumped slowly into refolding buffer (100 mM Tris, 2 mM Na-EDTA, 400 mM L-Arginine hydrochloride, 0.5 mM oxidized Glutathione in MilliQ water plus protease inhibitors) and further injections of 1 mg of HLA-A0201 heavy chain are made over 8 h intervals. The refolding process takes 48-72 h and approximately 20-30 % of the HLA-A0201 heavy chain is predicted to fold correctly with peptide and beta-2-microglobulin. The mix is then dialyzed thoroughly and the refolded MHC complexes are isolated using a ÄKTAFPLC fast protein liquid chromatography machine (GE Healthcare).

Generation of anti-HLA-A0201/peptide specific monoclonal hybridomas

The procedures we developed to generate the HLA-A0201/peptide specific monoclonals worked as follows (Supplementary Fig. 1): 5-6 weeks old BALB/c mice were immunized intraperitoneally with 25 μg of HLA-A0201/LMP1, HLA-A0201/LMP2A or HLA-A0201/EBNA1 monomers in complete Freund's adjuvant (Sigma-Aldrich) then boosted 3 times with 25 μg per boost in incomplete Freund's adjuvant (Sigma-Aldrich) over 45 days. The final boost at day 42 was intravenous (i.v). At day 45, the mice were euthanized and spleens removed aseptically. A single cell suspension of splenocytes was prepared by gentle homogenization of the tissue through 70 μm nylon mesh into 10 cm Petri dishes in HBSS (Invitrogen). The immune splenocytes were pulsed with 2 μg of biotinylated HLA-A0201/LMP1, HLA-A0201/LMP2A or HLA-A0201/EBNA1 monomer for 15 minutes at 4 °C followed by immunomagnetic selection using anti-biotin microbeads (Miltenyi Biotec). The selected splenocytes were fused using the protocol developed by Milstein and Kohler (ref. 32). The fused cells were washed and resuspended in HAT selection medium. 100 μl of fused cell suspension was cultured in microtitre plates for 3-5 days. Hybridoma clones were then scored macroscopically. Supernatants were collected over the next 21-days from the resulting hybridomas. The supernatants were tested by flow cytometry for the presence of HLA-A02/peptide responses using the C1R-A2 cell line pulsed with relevant peptide versus a control HLA-A02 restricted epitope derived from Influenza A (matrix epitope $M1_{58\text{-}66}$ GILGFVFTL).

Reference:

32. Kohler, G. & Milstein, C. Continuous cultures of fused cells secreting antibody of predefined specificity. *Nature* **256,** 495-497 (1975).

Supplementary figures legend

Supplementary Figure S1. Generation of TCR-like mAbs via enrichment of immunized splenocytes. Schematic of the novel procedure used for the production of TCR-like mAbs. This figure was drawn by P.A.M.

Supplementary Figure S2. Schematic of epitope determination based on calibration beads. Quantitation of epitope was carried out using QifiKit (Dako), the calibration beads consisting of pre-determined number of chemically bound isotype antibody. The beads were similarly treated with fluorochrome-conjugated antibody as cells of interest. Based upon MFI readings of the beads, a standard curve was generated. The number of epitope presented was inferred from this standard curve.

Supplementary Figure S3. Detection of mRNA and protein of EBNA1, LMP1 and LMP2A in CCRF-SB, RPMI-6666 and C666-1A2. (a) Visualization of PCR-amplified EBV latent gene products EBNA1, LMP1 and LMP2A from harvested mRNA of CCRF-SB, RPMI-6666 and C666-1A2 cell lines. The DNA ladder markers are as indicated. Beta-actin was used as a control. **(b)** Immunoblot analysis of EBNA1, LMP1 and LMP2A protein expression in CCRF-SB, RPMI-6666, and C666-1A2 cell lysates. The protein ladder markers are as indicated. Beta-actin was used as a control.

Supplementary Figure S4. Use of TCR-like monoclonal antibodies for visualization of EBV infected cells. Confocal microscopy visualization of staining of CCRF-SB, RPMI-6666 and C666-1A2 with the three TCR-like mAbs (red), anti-beta-2-microglobulin antibody (green) and DAPI (blue). The images were merged in the third column. Scale bars, 25 μm.

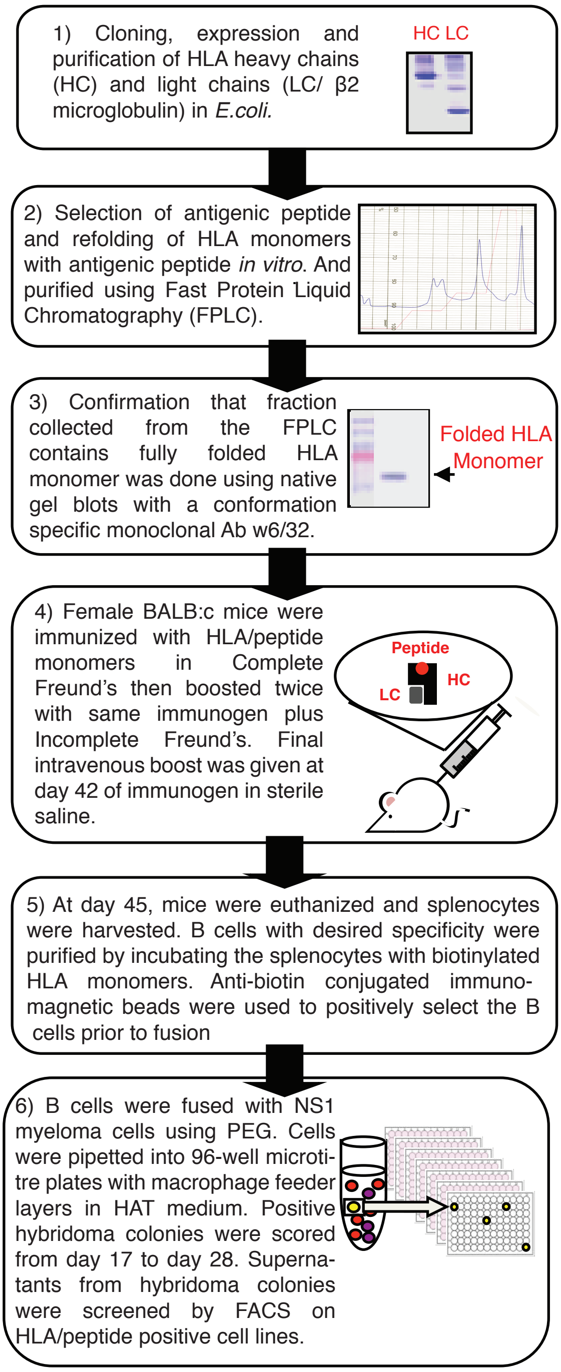
1) Cloning, expression and purification of HLA heavy chains (HC) and light chains (LC/ β2 microglobulin) in *E.coli.*
HC LC
2) Selection of antigenic peptide and refolding of HLA monomers with antigenic peptide *in vitro*. And purified using Fast Protein Liquid Chromatography (FPLC).
3) Confirmation that fraction collected from the FPLC contains fully folded HLA monomer was done using native gel blots with a conformation specific monoclonal Ab w6/32.
Folded HLA Monomer
4) Female BALB:c mice were immunized with HLA/peptide monomers in Complete Freund's then boosted twice with same immunogen plus Incomplete Freund's. Final intravenous boost was given at day 42 of immunogen in sterile saline.
Peptide
HC
LC
5) At day 45, mice were euthanized and splenocytes were harvested. B cells with desired specificity were purified by incubating the splenocytes with biotinylated HLA monomers. Anti-biotin conjugated immuno-magnetic beads were used to positively select the B cells prior to fusion
6) B cells were fused with NS1 myeloma cells using PEG. Cells were pipetted into 96-well microtitre plates with macrophage feeder layers in HAT medium. Positive hybridoma colonies were scored from day 17 to day 28. Supernatants from hybridoma colonies were screened by FACS on HLA/peptide positive cell lines.

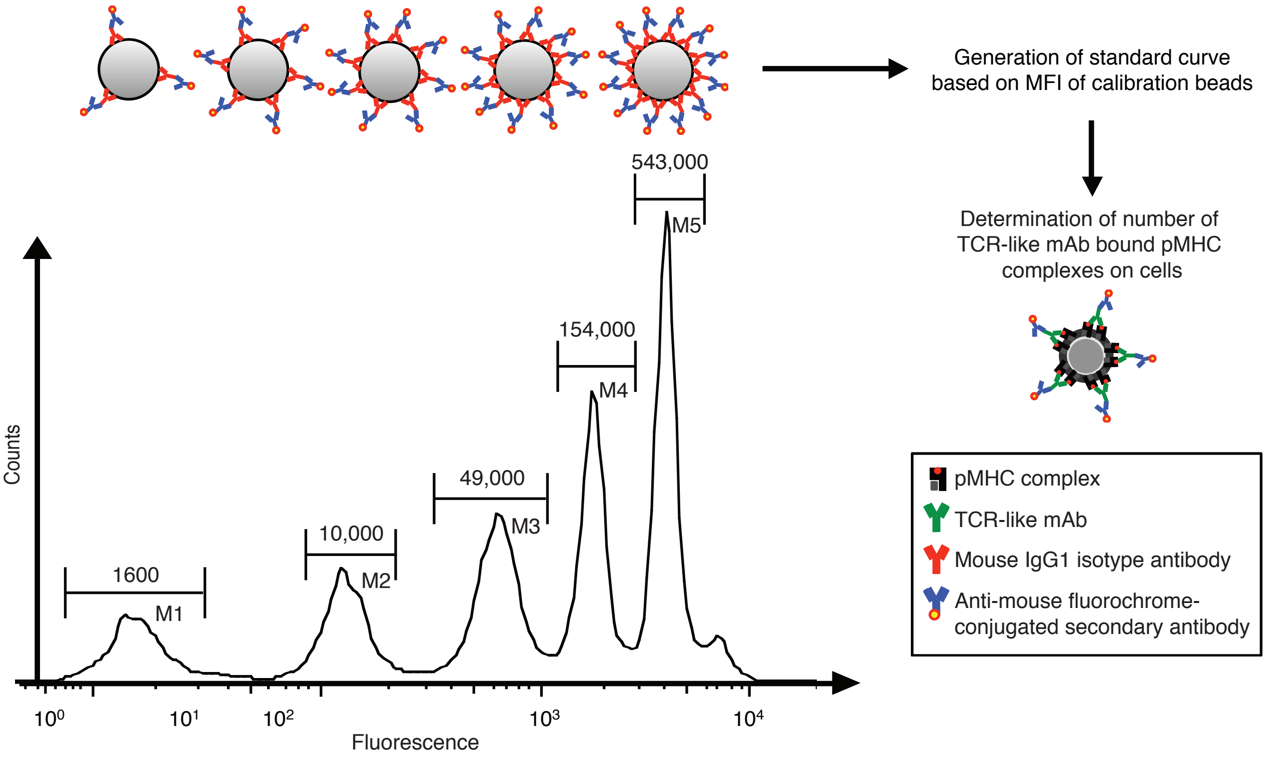
Generation of standard curve based on MFI of calibration beads
Determination of number of TCR-like mAb bound pMHC complexes on cells
543,000
M5
154,000
M4
49,000
M3
10,000
M2
1600
M1
Counts
Fluorescence
10^0
10^1
10^2
10^3
10^4
pMHC complex
TCR-like mAb
Mouse IgG1 isotype antibody
Anti-mouse fluorochrome-conjugated secondary antibody

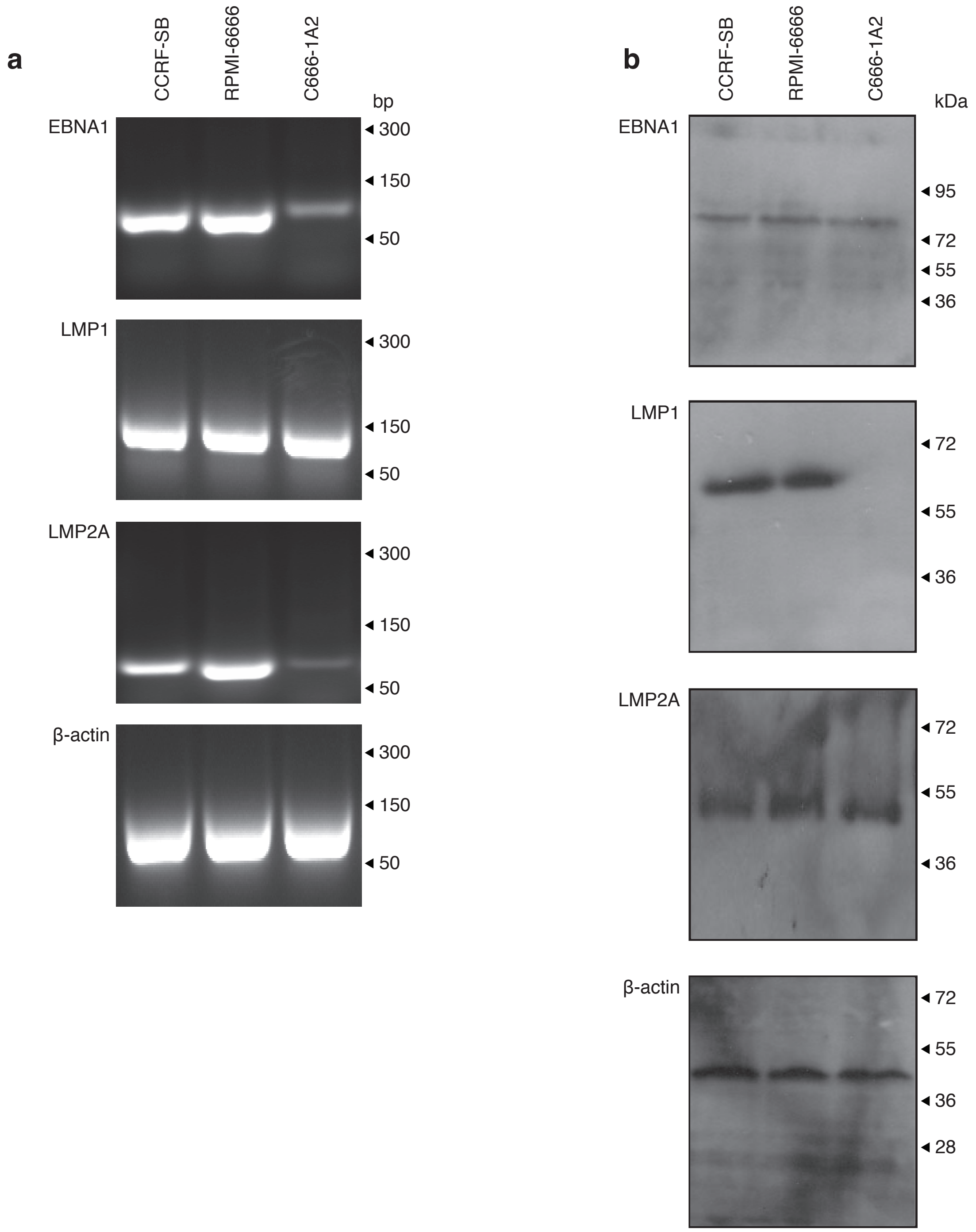
a
CCRF-SB
RPMI-6666
C666-1A2
bp
EBNA1
300
150
50
LMP1
300
150
50
LMP2A
300
150
50
β-actin
300
150
50
b
CCRF-SB
RPMI-6666
C666-1A2
kDa
EBNA1
95
72
55
36
LMP1
72
55
36
LMP2A
72
55
36
β-actin
72
55
36
28

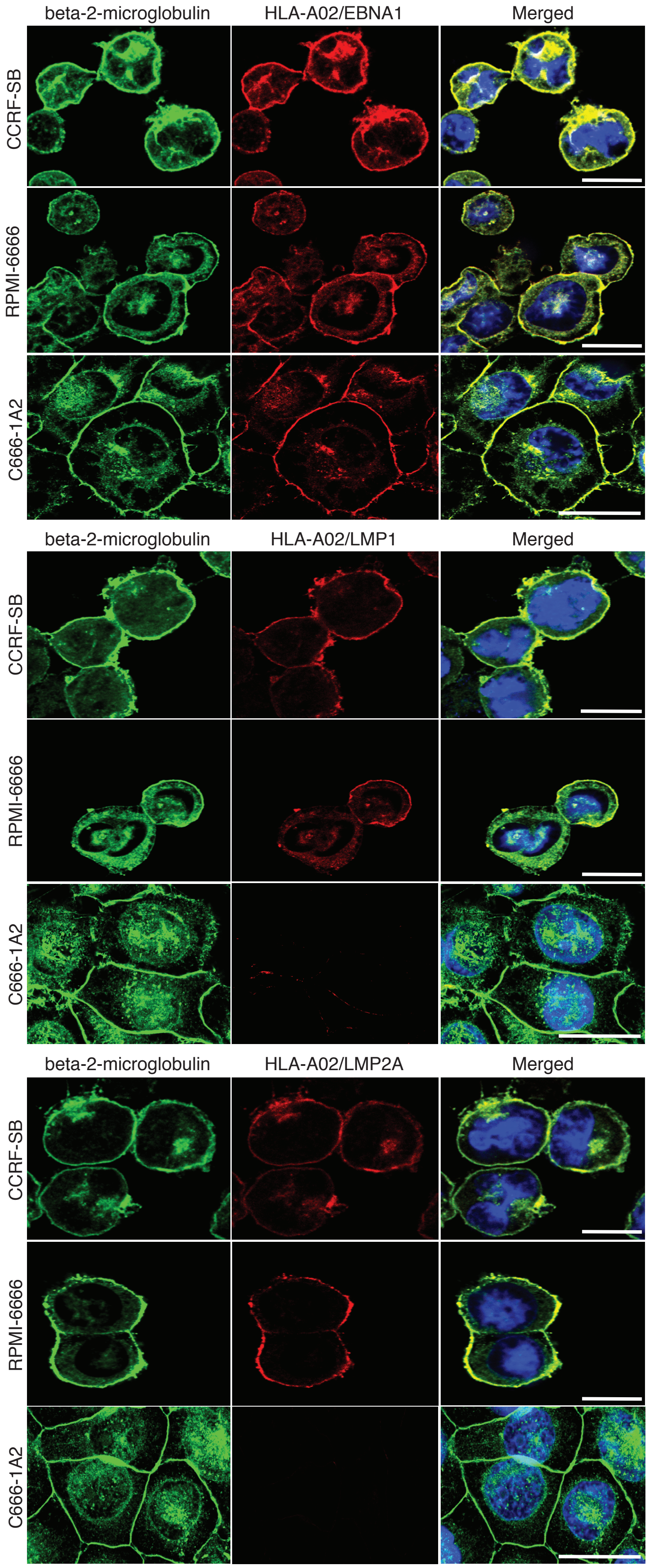
beta-2-microglobulin
HLA-A02/EBNA1
Merged
CCRF-SB
RPMI-6666
C666-1A2
beta-2-microglobulin
HLA-A02/LMP1
Merged
CCRF-SB
RPMI-6666
C666-1A2
beta-2-microglobulin
HLA-A02/LMP2A
Merged
CCRF-SB
RPMI-6666
C666-1A2
